# National and State Estimates of the Numbers of Adults and Children with Active Epilepsy — United States, 2015

**DOI:** 10.15585/mmwr.mm6631a1

**Published:** 2017-08-11

**Authors:** Matthew M. Zack, Rosemarie Kobau

**Affiliations:** 1 Division of Population Health, National Center for Chronic Disease Prevention and Health Promotion, CDC.

Epilepsy, a brain disorder leading to recurring seizures, has garnered increased public health focus because persons with epilepsy experience pronounced and persistent health and socioeconomic disparities despite treatment advances, public awareness programs, and expanded rights for persons with disabilities (*1*,*2*). For almost all states, epilepsy prevalence estimates do not exist. CDC used national data sources including the 2015 National Health Interview Survey (NHIS) for adults (aged ≥18 years), the 2011–2012 National Survey of Children’s Health (NSCH), and the 2015 Current Population Survey data, describing 2014 income levels, to estimate prevalent cases of active epilepsy, overall and by state, to provide information for state public health planning. In 2015, 1.2% of the U.S. population (3.4 million persons: 3 million adults and 470,000 children) reported active epilepsy (self-reported doctor-diagnosed epilepsy and under treatment or with recent seizures within 12 months of interview) or current epilepsy (parent-reported doctor-diagnosed epilepsy and current epilepsy). Estimated numbers of persons with active epilepsy, after accounting for income and age differences by state, ranged from 5,900 in Wyoming to 427,700 in California. NHIS data from 2010–2015 indicate increases in the number of persons with active epilepsy, probably because of population growth. This study provides updated national and modeled state-specific numbers of active epilepsy cases. Public health practitioners, health care providers, policy makers, epilepsy researchers, and other epilepsy stakeholders, including family members and people with epilepsy, can use these findings to ensure that evidence-based programs meet the complex needs of adults and children with epilepsy and reduce the disparities resulting from it.

Epilepsy has been assessed only intermittently in population surveys (*1*,*2*). Before 2010, the last U.S. national estimate of epilepsy prevalence was based on 1986–1990 data using one question assessing the occurrence of epilepsy or repeated seizures, convulsions, or blackouts in any household family members (*3*). Other recent estimates based on limited U.S. and international geographic regions, clinical samples, and decades-old data are not representative of the current U.S. population (*2*,*4*). Data from the 2010 and 2013 NHIS using a validated case definition indicate approximately 1% of the U.S. population had active epilepsy (*5*). A study using 2005 Behavioral Risk Factor Surveillance System data employing similar epilepsy case-ascertainment questions* provided state-level estimates of a history of epilepsy for 19 states (1.65%) and active epilepsy for 13 states (0.84%) (*6*). No substantial differences among states in the prevalence of a history of epilepsy or active epilepsy were detected (*6*). A third study, which extrapolated 2007–2011 administrative claims data from multiple states to the overall U.S. population found an epilepsy prevalence estimate of 0.84% (*4*). For almost all states, epilepsy prevalence estimates do not exist. Groups interested in reducing epilepsy prevalence need updated estimates of the numbers of persons living with epilepsy nationally and within their states. This study aims to provide updated national and modeled state-specific estimates of active epilepsy prevalence based on the latest data available to provide information for public health action to reduce epilepsy burden.

To estimate the number of prevalent cases of active epilepsy among adults aged ≥18 years, CDC analyzed three questions on epilepsy from the 2015 Sample Adult component of NHIS, an annual, cross-sectional household survey of the civilian, noninstitutionalized U.S. population. Adults classified as having “active epilepsy” reported a history of doctor-diagnosed epilepsy and were taking medication to control it, had had one or more seizures in the past year, or both (Table 1) (*5*,*6*). Validation of survey questions for surveillance of active epilepsy yielded sensitivity and specificity exceeding 80% and 99%, respectively, with a positive predictive value of 74% similar to validation estimates seen in surveillance of other chronic disorders (*5*). Only 0.07% of adults in 2015 refused to answer or did not know if they had doctor-diagnosed active epilepsy. To estimate prevalent cases of active epilepsy among children aged 0–17 years, CDC analyzed data from the 2011–2012 NSCH,^†^ a cross-sectional telephone survey of households with at least one resident child aged 0–17 years at interview. NSCH asks parents or guardians if a doctor or health care provider ever told them that their child had epilepsy or seizure disorder, and if so, if their child currently has epilepsy or seizure disorder (current epilepsy) (Table 1). Only 0.03% of parents or guardians refused to answer or did not know if a doctor had ever told them their child had epilepsy or a seizure disorder. Prevalence of current epilepsy among children based on NSCH data was estimated to be 6.3 per 1,000, similar to estimates from administrative data (*7*,*8*).

**TABLE 1:** Epilepsy surveillance case ascertainment questions, by survey

Survey	Questions	Possible responses
**National Health Interview Survey (2015)**	1. Have you ever been told by a doctor or other health professional that you have a seizure disorder or epilepsy?	1) Yes, 2) No, 7) Refused, 8) Not ascertained, 9) Don’t know
	2. Are you currently taking any medicine to control your seizure disorder or epilepsy?	1) Yes, 2) No, 7) Refused, 8) Not ascertained, 9) Don’t know
	3. Today is <date>. Think back to last year about the same time. About how many seizures of any type have you had in the past year?	0) None, 1) One, 2) Two or three, 3) Between four and ten, 4) More than 10, 7) Refused, 8) Not ascertained, 9) Don’t know
**National Survey of Children’s Health (2011–2012)**	1. Has a doctor or health care provider ever told you that your child has epilepsy or a seizure disorder?	1) Yes, 2) No, 7) Refused, 8) Not ascertained, 9) Don’t know
	2. Does your child currently have epilepsy or a seizure disorder?	1) Yes, 2) No, 7) Refused, 8) Not ascertained, 9) Don’t know

Obtaining state-level estimates required using the best available data to confirm that epilepsy prevalence did not differ significantly across states (*6*). Epilepsy prevalence and state populations do differ by age and income distribution. NHIS and NSCH data was used to calculate the prevalence (proportion) of active epilepsy for three age groups (0–17 years, 18–64 years, and ≥65 years) stratified by three family income groups (0%–99%, 100%–199%, ≥200% of poverty thresholds). Data for 2014 was obtained for the three age groups and three family income groups among civilian and military noninstitutionalized populations for each state from the U.S. Census's Current Population Survey 2015 Annual Social and Economic Supplement.^§^ Multiplying the age- and income-specific active epilepsy prevalence estimates by the population estimates for each of the three age and income groups yielded state-level estimates of active epilepsy, indirectly standardized for age and income.^¶^ Adding these standardized estimates for both groups from each data set produced total estimated numbers of cases with active epilepsy. Combining the variance estimates of both adults and children with epilepsy from each survey and of these age- and income-specific population estimates as the variance of the product of these two random variables yielded 95% confidence intervals for these total estimates.**

In 2015, 1.2% (95% confidence interval = 1.1–1.4) of the U.S. population was classified as having active epilepsy (3.4 million; 3 million adults and 470,000 children). Among adults, the estimated number of cases of active epilepsy ranged from 5,100 in Wyoming to 367,900 in California (Table 2). Among children, the estimated number of cases of current epilepsy ranged from 800 in Wyoming to 59,800 in California. The number of persons estimated to have active epilepsy was <14,000 in nine states and the District of Columbia, 14,000–32,799 in 11 states, 32,800–56,799 in nine states, 56,800–92,699 in 10 states, and ≥92,700 persons in 11 states. (Table 2).

**TABLE 2:** Estimated numbers of active epilepsy cases, by state and age group — United States, 2015

Geographic area	Age group (yrs)		
	All ages	<18*	≥18^†^
	No. (95% CI^§^)	No. (95% CI)	No. (95% CI)
**United States**	**3,439,600 (3,009,100–3,870,100)**	**471,900 (392,600–551,200)**	**2,967,700 (2,544,500–3,390,800)**
Alabama	54,100 (46,400–61,900)	7,500 (5,900–9,200)	46,600 (39,000–54,200)
Alaska	7,200 (6,100–8,300)	1,100 (800–1,400)	6,100 (5,000–7,200)
Arizona	77,000 (66,400–87,500)	11,200 (8,900–13,600)	65,700 (55,400–76,000)
Arkansas	32,800 (28,000–37,600)	4,900 (3,700–6,100)	28,000 (23,300–32,600)
California	427,700 (372,600–482,900)	59,800 (49,000–70,600)	367,900 (313,800–422,000)
Colorado	56,800 (48,300–65,300)	7,800 (6,000–9,600)	49,000 (40,700–57,300)
Connecticut	35,900 (30,400–41,400)	4,500 (3,400–5,700)	31,400 (26,000–36,800)
Delaware	9,700 (8,200–11,100)	1,300 (900–1,600)	8,400 (7,000–9,900)
District of Columbia	7,500 (6,300–8,800)	800 (600–1,100)	6,700 (5,500–7,900)
Florida	223,900 (194,100–253,800)	27,300 (21,900–32,800)	196,600 (167,200–225,900)
Georgia	110,200 (94,900–125,500)	16,700 (13,200–20,100)	93,500 (78,600–108,500)
Hawaii	14,000 (11,900–16,100)	2,000 (1,500–2,400)	12,000 (10,000–14,100)
Idaho	16,800 (14,200–19,300)	2,600 (2,000–3,200)	14,200 (11,700–16,600)
Illinois	136,600 (117,900–155,400)	18,600 (14,900–22,400)	118,000 (99,700–136,400)
Indiana	69,500 (59,600–79,400)	10,600 (8,300–13,000)	58,900 (49,200–68,500)
Iowa	31,400 (26,800–36,100)	4,400 (3,400–5,400)	27,000 (22,500–31,600)
Kansas	29,900 (25,500–34,300)	4,400 (3,400–5,400)	25,500 (21,200–29,900)
Kentucky	49,500 (42,000–57,000)	6,800 (4,900–8,700)	42,700 (35,500–50,000)
Louisiana	54,900 (46,600–63,200)	7,900 (6,200–9,700)	47,000 (38,900–55,100)
Maine	14,100 (11,900–16,300)	1,700 (1,200–2,200)	12,400 (10,300–14,600)
Maryland	59,900 (50,700–69,100)	7,900 (6,200–9,700)	52,000 (42,900–61,000)
Massachusetts	71,600 (60,900–82,300)	8,400 (6,500–10,300)	63,200 (52,600–73,700)
Michigan	108,900 (93,300–124,500)	13,600 (10,800–16,400)	95,300 (79,900–110,600)
Minnesota	53,700 (45,700–61,700)	7,400 (5,900–9,000)	46,300 (38,400–54,100)
Mississippi	35,700 (30,600–40,700)	5,100 (3,900–6,300)	30,600 (25,700–35,500)
Missouri	61,200 (52,400–70,000)	8,300 (6,500–10,100)	52,900 (44,200–61,600)
Montana	10,800 (9,100–12,600)	1,400 (1,000–1,800)	9,400 (7,700–11,100)
Nebraska	19,600 (16,600–22,500)	2,800 (2,200–3,500)	16,700 (13,800–19,600)
Nevada	31,600 (26,800–36,400)	4,400 (3,300–5,400)	27,200 (22,500–31,900)
New Hampshire	13,100 (11,100–15,200)	1,500 (1,100–1,900)	11,600 (9,600–13,700)
New Jersey	92,700 (79,100–106,200)	12,000 (9,500–14,500)	80,600 (67,300–93,900)
New Mexico	23,200 (19,800–26,500)	3,400 (2,600–4,200)	19,800 (16,400–23,100)
New York	215,200 (186,300–244,000)	26,600 (21,600–31,500)	188,600 (160,200–217,100)
North Carolina	110,100 (94,700–125,500)	15,200 (11,800–18,500)	94,900 (79,900–110,000)
North Dakota	7,300 (6,200–8,500)	1,000 (700–1,200)	6,400 (5,300–7,500)
Ohio	126,400 (109,300–143,400)	16,900 (13,600–20,300)	109,400 (92,700–126,200)
Oklahoma	41,100 (34,900–47,300)	6,400 (5,000–7,900)	34,700 (28,700–40,700)
Oregon	42,900 (36,300–49,400)	5,400 (4,100–6,800)	37,400 (31,000–43,900)
Pennsylvania	133,000 (114,600–151,400)	16,900 (13,500–20,200)	116,100 (98,000–134,200)
Rhode Island	11,100 (9,300–12,900)	1,300 (900–1,700)	9,800 (8,100–11,500)
South Carolina	53,400 (45,500–61,300)	7,100 (5,500–8,700)	46,300 (38,500–54,000)
South Dakota	8,900 (7,400–10,400)	1,300 (900–1,600)	7,600 (6,200–9,100)
Tennessee	73,900 (62,900–84,800)	10,000 (7,800–12,300)	63,800 (53,100–74,600)
Texas	292,900 (255,400–330,300)	47,200 (38,500–56,000)	245,600 (209,200–282,000)
Utah	29,300 (24,900–33,600)	5,300 (4,100–6,500)	24,000 (19,800–28,200)
Vermont	6,300 (5,300–7,300)	700 (500–900)	5,600 (4,700–6,600)
Virginia	84,800 (72,600–97,000)	11,000 (8,800–13,200)	73,800 (61,800–85,800)
Washington	74,600 (64,000–85,200)	10,200 (8,100–12,300)	64,400 (54,000–74,800)
West Virginia	21,500 (18,100–25,000)	2,500 (1,900–3,100)	19,000 (15,600–22,500)
Wisconsin	59,600 (50,800–68,300)	7,900 (6,300–9,500)	51,700 (43,100–60,300)
Wyoming	5,900 (5,000–6,800)	800 (600–1,000)	5,100 (4,200–6,000)

**Abbreviation:** CI = confidence interval.

* Active epilepsy cases in children are estimated from the current epilepsy prevalence in children (2011–2012 National Survey of Children's Health) and the population of children, accounting for the ratios of family income to poverty thresholds.

^†^ Active epilepsy cases in adults are estimated from the prevalence of active epilepsy (taking medication, having had a seizure in the past year, or both) in adults (2015 National Health Interview Survey) and the population of adults, accounting for the ratios of family income to poverty thresholds. The total population estimates come from the 2014 weighted person counts of the Current Population Survey, 2015 Annual Social and Economic Supplement of the civilian noninstitutionalized population living in houses and military population living in houses.

^§^ Confidence interval represents only sampling uncertainty from the sampling uncertainties in the prevalence estimates and in the state-specific and age-specific ratios of family income to poverty thresholds.

## Discussion

This study provides updated national and estimated state-specific numbers of the active epilepsy cases. Affecting 3.4 million U.S. residents, epilepsy is not a rare condition. Epilepsy poses substantial individual and societal burdens that require heightened public health action (*1*,*2*). As a complex condition varying in severity and impact, it affects persons of all ages and racial and ethnic groups, especially those with the lowest incomes (*2*,*5*,*9*). Persons with epilepsy often have multiple co-occurring conditions (e.g., stroke, heart disease, depression, or developmental delay) that complicate their epilepsy management, impair life goals, and contribute to early mortality (*1*,*2*). Among five chronic conditions in children and adolescents selected because of their adverse impact on academic and health outcomes, epilepsy is the costliest and the second most common (*8*). Children with seizures are more likely to live in poverty, and their parents more frequently report food insecurity (*9*). Direct yearly health care costs per person with epilepsy ranged from $10,192 to $47,862 (2013 U.S. dollars) and were higher for persons with uncontrolled seizures (*10*).

Medicaid recipients have a higher prevalence of epilepsy, especially among adults aged 20–64 years (3.4%) (*4*); this study adjusted for income to account for this confounder. The estimated 3 million U.S. adults with active epilepsy and 470,000 U.S. children with current epilepsy in 2015 exceed the estimated 2.3 million U.S. adults in 2010 (*5*) and the 450,000 U.S. children with current epilepsy in 2007 (*7*). The estimated increase in numbers of persons with epilepsy is not explained by age or income, because this study controlled for these known confounders. The increase is likely because of population growth over the past decade, or other unknown factors (e.g., an increased willingness to disclose one has epilepsy). The number of prevalent cases of active epilepsy by state generally mirrors the states’ population distributions. The 2015 NHIS epilepsy prevalence estimate (1.2%) in this study is roughly consistent with the BRFSS estimate from 13 states (0.84% [95% confidence interval = 0.74–0.96]) that used a slightly more conservative approach assessing a 3-month seizure recall period versus 12 months (*6*).

The findings in this report are subject to at least four limitations. First, because these estimates depend on self-report, they might be subject to reporting bias. Second, these state estimates do not account for possible differences in seizure type, severity, or etiology. Third, underreporting associated with perceived repercussions in disclosing epilepsy (e.g., stigma or driver’s license restrictions) (*2*) and the exclusion of institutionalized adults from the NHIS and the Census might underestimate epilepsy prevalence. Fourth, the assumption of applying national estimates to states is based on findings from 13 geographically disparate states indicating no differences in epilepsy prevalence, after accounting for multiple comparisons and sample size limitations (*6*). Although adjusting for age and income might account for some of the variation in prevalence across all states in this study, without available direct surveillance data on epilepsy, these estimates of active epilepsy cases in states need empirical confirmation.

Public health practitioners, health care providers, policy makers, epilepsy researchers, and other epilepsy stakeholders, including family members and people with epilepsy, can use these findings to ensure that evidence-based programs meet the complex needs of adults and children with epilepsy and reduce the disparities resulting from it.

**Summary**

**What is already known about this topic?**

Epilepsy is a common neurologic disorder resulting in substantial health, social, and mortality disparities.

**What is added by this report?**

In 2015, approximately 3 million U.S. adults and 470,000 children had active epilepsy. For almost all states, epilepsy prevalence estimates do not exist. Estimated numbers of active epilepsy ranged from 5,900 persons with epilepsy in Wyoming to more than 427,000 in California. The number of persons with active epilepsy increased compared with earlier years, likely because of population growth.

**What are the implications for public health practice?**

This study provides updated national estimates and the first modeled estimates of active epilepsy cases for all States. Public health practitioners, health care providers, policy makers, epilepsy researchers, and other epilepsy stakeholders including family members and people with epilepsy, can use these findings to ensure that evidence-based programs meet the complex needs of adults and children with epilepsy and reduce the disparities resulting from it.

## Footnotes

* In 2005, the Behavioral Risk Factor Surveillance System included the following five epilepsy questions: 1) “Have you ever been told by a doctor that you have a seizure disorder or epilepsy?” (response options: “yes,” “no,” “don't know,” and “refused”). Participants who answered “yes” to this question were asked some or all of the following questions: 2) “Are you currently taking any medicine to control your seizure disorder or epilepsy?” (response options: “yes,” “no,” “don't know,” and “refused”); 3) “How many seizures have you had in the last 3 months?” (response options: “none,” “one,” “more than one,” “no longer have epilepsy or seizure disorder,” “don't know,” and “refused”); 4) “In the past year, have you seen a neurologist or epilepsy specialist for your epilepsy or seizure disorder?” (response options: “yes,” “no,” “don't know,” and “refused”); and 5) “During the past 30 days, to what extent has epilepsy or its treatment interfered with your normal activities like working, school, or socializing with family or friends?” (response options: “not at all,” “slightly,” “moderately,” “quite a bit,” “extremely,” “don't know,” and “refused”). The only change between the earlier BRFSS and the later NHIS case-ascertainment is that BRFSS includes a 3-month recall for any seizure occurrence and NHIS, a 12-month recall for any seizure occurrence. This change reflected updated consensus guidance on case-ascertainment for community-based epilepsy surveillance.

† National Survey of Children’s Health, 2011–2012. https://www.cdc.gov/nchs/slaits/nsch.htm.

§ Current Population Survey Annual and Social Economic Supplement, 2014 Poverty Status by State and Age Groups. https://www.census.gov/data/tables/time-series/demo/income-poverty/cps-pov/pov-46.2014.html.

¶ The estimated numbers of active epilepsy cases are calculated in the same way as the expected numbers in indirect standardization are calculated to account for confounding (https://www.ncbi.nlm.nih.gov/pmc/articles/PMC3406211).

** The formula to calculate the variances for the 95% confidence intervals of the expected numbers of active epilepsy cases is found in http://www.jstor.org/stable/2281592.
